# Circulating anti-citrullinated peptide antibodies, cytokines and genotype as biomarkers of response to disease-modifying antirheumatic drug therapy in early rheumatoid arthritis

**DOI:** 10.1186/s12891-015-0587-1

**Published:** 2015-05-29

**Authors:** Mahmood M. T. M. Ally, Bridget Hodkinson, Pieter W. A. Meyer, Eustasius Musenge, Gregory R. Tintinger, Mohammed Tikly, Ronald Anderson

**Affiliations:** Department of Internal Medicine Faculty of Health Sciences, University of Pretoria, Bophelo Road, Private Bag X663, Pretoria, 0001 South Africa; Medical Research Council Unit for Inflammation and Immunity, Department of Immunology, Faculty of Health Sciences, University of Pretoria, Bophelo Road, Pretoria, 0001 South Africa; Division of Rheumatology, Chris Hani Baragwanath Academic Hospital, Faculty of Health Sciences, University of the Witwatersrand, Chris Hani Road, Johannesburg, 2013 South Africa; Tshwane Academic Division of the National Health Laboratory Service, Bophelo Road, Pretoria, 0001 South Africa; Biostatistics and Epidemiology Division, School of Public Health, Faculty of Health Sciences, University of the Witwatersrand, York Road, Johannesburg, 2193 South Africa

**Keywords:** Anticyclic citrullinated peptide antibodies, Cytokines, Shared epitope, Disease modifying antirheumatic drugs, Rheumatoid arthritis

## Abstract

**Background:**

To measure circulating anti-citrullinated peptide antibodies (ACPA) and cytokines pre- and 6 months post-therapy as a strategy to predict and optimize responses to traditional disease-modifying antirheumatic drugs (DMARDs) in early RA, which is an unmet need in developing countries.

**Patients and methods:**

A cohort of 140 predominantly (88.5 %) black female South African patients with early RA was treated with synthetic DMARDs, mostly methotrexate (MTX) alone, or in combination with low-dose oral corticosteroids (CS). Circulating ACPA and a panel of circulating cytokines/chemokines/growth factors were measured at baseline and after 6 months of therapy in relation to disease activity and Shared Epitope (SE).

**Results:**

Following 6 months of therapy, the median simplified disease activity index (SDAI) declined from a baseline of 41.4 to 16.0 (*p* = 0.0001) for the entire cohort, which was paralleled by significant falls in median serum ACPA levels (516.6 vs. 255.7 units/ml, *p* = <0.0001) and several of the circulating cytokines (IL-4, IL-7, IL-8, G-CSF, VEGF; p < 0.0010 – p < 0.0001) which were most evident in the subgroup of patients treated with a combination of MTX and CS. Although biomarker concentrations decreased most notably in the low-disease activity group post-therapy, no significant correlations between these biomarkers and disease activity were observed, Baseline ACPA levels, but not SDAI or cytokines, were significantly higher in the subgroup of risk allele-positive patients (561.1 vs. 331.9 units/ml, p < 0.05), while no associations with ACPA and a smoking history were evident.

**Conclusions:**

The use of DMARDs in RA is associated with significant decreases in ACPA and cytokines which did not correlate with changes in SDAI, precluding the utility of serial measurement of these biomarkers to monitor early responses to therapy, but may have prognostic value.

**Electronic supplementary material:**

The online version of this article (doi:10.1186/s12891-015-0587-1) contains supplementary material, which is available to authorized users.

## Background

Raised levels of anti-citrullinated peptide antibodies (ACPA) levels have diagnostic and prognostic value, and have been incorporated in the 2010 Eular/ACR rheumatoid arthritis (RA) classification criteria [1]. Studies investigating therapy-associated alterations in ACPA levels in patients with early RA have focused predominantly on biologic disease-modifying anti-rheumatic drugs (DMARDs) [2]. However, the association of a decrease in ACPA levels with therapeutic response has been variable [3–13]. On the other hand, raised ACPA levels may account for relapse and persistence of disease, with the magnitude of the pre-therapy levels being inversely associated with response to methotrexate (MTX) in early undifferentiated arthritis [14]. ACPA levels have not only been shown to correlate with response to anti-TNF therapy, but are also predictive of response to rituximab [15].

Cytokines play an integral role in the pathogenesis of RA and their importance as therapeutic targets is well established. However, the utility of serial measurement of circulating cytokines in RA is not clearly defined. Changes in cytokine levels post-therapy, especially the balance between pro- and anti-inflammatory cytokines, have the potential to aid in monitoring treatment response, guide future therapy and/or have prognostic implications [16]. For example, a decrease in IL-7 levels after treatment with MTX has been found to correlate with improved clinical measures of disease activity [17]. In addition, TNF levels below 20.1 pg/ml have been shown to be associated with a good response to MTX, while a low IL-2 level at baseline is an independent predictor of response to synthetic DMARDs [18]. IL-6 levels greater than 4.03 pg/ml post-treatment with MTX have been associated with radiographic progression [19]. The pre-treatment levels of cytokines may also be predictive of response to biologic DMARDs. Patients with elevated serum TNF levels may require higher doses of infliximab, while high levels of IL-17 are possibly predictive of a subgroup of RA patients resistant to TNF blockade [20]. Cytokine ratios may also have prognostic significance, with the IL-6/IL-10 ratio being associated with new coronary events in the general population [21].

The “shared epitope” (SE) is a well-recognized genetic risk factor for, and poor prognostic marker in RA, being associated with both ACPA positivity and a poorer response to MTX monotherapy [11, 22–25]. Patients who do not carry the risk alleles generally have milder disease, less radiographic progression and are more likely to respond to DMARDs.

Most studies focused on genotype and profiling of circulating immune biomarkers in prediction of risk and response to therapy in patients with RA have been undertaken in developed world countries. However, RA in the developing world, where there is often little-or-no access to expensive biologic therapies, is associated with as much, if not more, morbidity, than in developed countries, underscoring the importance of discerning clinical utility of traditional DMARD-based therapy in limited resource settings. To our knowledge, measurement of the SE/risk allele status and its association with longitudinal alterations in clinical disease activity, as well as the concentrations of circulating biomarkers of immune activation, specifically autoantibodies, acute phase reactants and cytokines/chemokines following initiation of DMARD-based therapy has not been described in black African patients with early RA.

Accordingly, the objectives of the present study were to: i) characterize changes in ACPA and cytokines in relation to disease activity following synthetic DMARD therapy in an early RA cohort of predominantly black South African patients; and ii) evaluate changes in disease activity, APCA and cytokines in relation to genotype in the different treatment responder groups.

## Methods

After obtaining approval from the Research Ethics Committees of the Faculties of Health Sciences of the University of Pretoria and the University of the Witwatersrand 140 South African patients who met the 1987 revised classification criteria for RA and were DMARD-naïve, attending two public sector rheumatology clinics, were enrolled as part of the prospective observational Gauteng Rheumatoid Early Arthritis (GREAT) study [26, 27]. Informed written consent was obtained from all participants before recruitment into the study. Patients were followed as per routine standard of care, with the attending clinicians deciding on the type of DMARD therapy and frequency of follow-up visits. In all, 43 patients were treated with MTX monotherapy, 33 with MTX in combination with prednisone; 9 on a combination of MTX and chloroquine (CQ), 1 on sulfasalazine (SZP), 1 on CQ monotherapy, while the remainder were on varying combinations of all 3 DMARDs without or with prednisone. The initial dose of MTX was 7.5-15 mg/week [mean (SD) dose 13.1 (3.2) mg/week], gradually increasing to a maximum of 25 mg/week. In 77(55 %) patients low-dose prednisone, 5–7.5 mg daily, was co-prescribed.

Data collected at baseline and 6 months included the simple disease activity index (SDAI) [28], rheumatoid factor (RF), ACPA and a panel of cytokines, chemokines and growth factors.

### Assessment of clinical disease activity

The SDAI was used as the measure of disease activity as this is a validated measure often used in routine clinical practice.

Subgroup analysis included assessment of SDAI categories and SDAI responder groups at 6 months. The levels of disease activity were categorised as follows: i) SDAI ≤ 11 (low-disease activity, LDA); ii) SDAI ≤ 26 (moderate-disease activity, MDA); and iii) SDAI > 26 (high-disease activity, HDA). SDAI response was categorized according to percentage improvement from baseline SDAI with <50 % being no response, ≥ 50 < 70 % a minor response, ≥70 < 85 % a moderate response and ≥85 % a major response [28].

### Laboratory investigations

ACPA were measured by immunofluorimetry using the Immunocap 250 system based on second generation CCP as antigen and reagents and controls provided by the manufacturer (Phadia AB, Uppsala, Sweden). According to the manufacturer the intra-run and inter-run coefficients of variation (CVs) are 5.1-10.5 % and 2.6-7.7 % respectively, with values of >10 U/ml deemed positive [26, 27].

C-reactive protein (CRP) and rheumatoid factor (RF, composite IgM, IgG, IgA) were assayed by immunonephelometry (Siemens Health Care Diagnostics, BN Prospec Nephelometer, Newark, USA) using reagents and controls supplied by the manufacturer with results considered positive when they exceeded values of 5 μg/ml and 11 IU/ml respectively [26, 27].

#### Cytokines/chemokines

Circulating cytokines and their antagonists, representative of various types of immune, inflammatory and structural cells, were assayed using Bio-Plex suspension multiplex suspension bead array technology Bio-Rad Laboratories Inc., Hercules CA, USA) using standards and controls supplied by the manufacturer for setting up an 8 point standard curve as reference, as well as validating the assay with a low and high control serum for each of the 17 analytes. [26, 27]. Intra-run variability was monitored by introducing 5 previously measured samples with a CV of <5 % achieved. Inclusion of a blank bead suspension was used to control for non-specific binding. Results are expressed as picograms (pg)/ml. Cytokines/chemokines analysed included IL-1β, IL-1Ra, IL-4, IL-5, IL-6, IL-7, IL-8, IL-10, IL-12, IL-13, IL-17, G-CSF, GM-CSF, IFN-γ, TNF, VEGF, CCL2 and CCL4. Ratios of IL-1/IL-1Ra, IL 17/IL 10, IFN- γ/IL- 4, IL-8/IL-10 and IL-6/IL-10 were also calculated, albeit in smaller groups of patients.

#### Genotyping

Genotyping of HLA-DRB1 alleles was performed using a DNA-based high-resolution typing method, LABType®HD DRB1 (One Lambda Inc, Canoga Park, California, USA), utilizing reverse sequence specific oligonucleotide (rSSO) Luminex xMAP® technology probes; SE classification done according to the Du Montcel classification [29], with the groups pooled according to SE status into risk-allele-positive (S2,S3P) and -negative (S1,S3D,X) subgroups [30].

### Statistical analysis

The demographic, clinical and biomarker data are described using frequencies and percentages for categorical types such as gender. Medians and ranges were used to describe the skewed numerical clinical and biomarker data. The Mann–Whitney test was used to assess differences in medians for two independent groups, while the Wilcoxon signed-rank test was used to compare two dependent groups (i.e., baseline vs. six months). The Kruskal-Wallis test was used to determine differences in medians of more than three independent groups. A p-value of less than 0.05 was deemed significant, and, where appropriate, Bonferroni corrections to the p-values for multiple tests were applied. Spearman’s pairwise correlations were used to find the strengths of association between circulating cytokines and reduction of SDAI at 6 months. Multivariable analyses were performed using backward stepwise logistic regression on the outcome of disease activity at 6 months and any baseline clinical and laboratory variables with a p-value ≤ 0.1 in the univariate analysis were included in the models.

## Results

### Patient demographics

The baseline demographic, clinical features and laboratory indices of disease activity, with the exception of cytokines/chemokines, for the entire cohort are shown in Table 1 and Additional file 1: Table S1.

**Table 1:** Demographics of patients (*n* = 140)

	n or median	% or IQR
Black ethnicity (%)	124.0	88.5
Females (%)	110.0	79.2
Age in years, median (IQR)	47.8	14.4
Symptom duration in months, median (IQR)	9.7	11.4
Smoking history (%)	34.0	24.2
Nodulosis (%)	27.0	19.3
Rheumatoid factor positive (%) IU/ml	114.0	81.4
ACPA [n at baseline = 126] (%) U/ml	106.0	84.1
Shared Epitope positive [n at baseline =115] (%)	106.0	92.1
CRP baseline,median (IQR) μg/ml	17.1	42.3
SDAI baseline, median (IQR)	41.4	24.1

### Changes in SDAI and biomarker concentrations after 6 months of therapy

These results for SDAI and those biomarkers which decreased significantly in concentration following DMARD therapy are shown in Table 2 (data of all biomarker changes post-therapy shown in Additional file 1: Table S2). The median SDAI declined from 41.4 (IQR 24.1) at baseline to 16 (IQR 15.8) (*p* = 0.0010. The categories of response were: i) no response: *n* = 58 (41.4 %); ii) minor response: *n* = 40 (28.5 %); iii) moderate response: *n* = 22 (15.7 %); and iv) major response: *n* = 20 (14.1 %) (Additional file 1: Table S3). These changes coincided with significant decreases in the median serum concentrations of ACPA and several cytokines (IL-4, IL-7, IL-8, G-CSF, VEGF), as well as the ratios of IL-1β/IL-1Ra and IL-17/IL-10 (p < 0.05). Although significantly decreased in the entire cohort, the levels of circulating IL-7, IL-8, G-CSF and VEGF remained unchanged or increased at 6 months in 41.4 %, 30.1 %, 43.9 % and 42.2 % of patients respectively. However, the magnitudes of the decrements in the concentrations of these biomarkers did not correlate significantly with the improvement in SDAI or its individual components of tender and swollen joint counts (SJC), C-reactive protein (CRP), physician and patient global assessments.

**Table 2:** Significant changes in SDAI scores and circulating biomarker concentrations following 6 months DMARD therapy

Variable	n	Baseline		6 months		P value
		Median	IQR	Median	IQR	
SDAI	140	41.39	24.10	16 .00	15.81	0.0001
CRP (μg/ml)	140	17.10	42.25	8.90	17.35	0.0001
ACPA (IU/ml)	100	516.60	1027.75	255.65	677.20	0.0001
IL-4 (pg/ml)	32	4.70	10.28	1.51	6.53	0.0075
IL-7 (pg/ml)	123	20.32	82.22	16.69	23.46	0.0013
IL-8 (pg/ml)	123	8.79	12.65	5.70	7.09	0.0001
G-CSF (pg/ml)	123	13.82	60.02	7.23	51.61	0.0083
VEGF (pg/ml)	123	168.56	441.33	92.62	167.21	0.0010
Ratio IL-1 / IL-1Ra	37	0.05	0.05	0.03	0.06	0.0130
Ratio IL-17 / IL-10	30	10.42	15.61	3.72	3.81	0.0001

Only those biomarkers which decreased significantly post-therapy are shown

ACPA levels decreased in the majority of seropositive cases, 66 (85.7 %), and in a small minority ACPA levels actually increased, 11 (14.2 %), while seroconversion and seroreversion were noted in 3 and 8 patients, respectively, following 6 months of therapy. The fall in ACPA levels was greatest in patients with higher baseline values (data not shown). There were no associations between alterations in ACPA post-therapy with either the baseline SDAI or SDAI at 6 months. In the case of smokers, there were no differences in the levels of ACPA at baseline or the magnitudes of the decrements thereof after 6 months between patients with a positive or negative history of ever having smoked.

### Comparison of pre- and post-therapy alterations in SDAI values and biomarker levels in patients treated with methotrexate (MTX) without and with prednisone

Subgroup analysis of significant changes in SDAI scores and circulating biomarkers, stratified by type of DMARD therapy (MTX monotherapy vs. the combination of MTX and prednisone) at 6 months, are shown in Table 3. Pre-therapy SJC (*p* = 0.0458), CRP (*p* = 0.0250) and IL-7 (*p* = 0.0110) levels were significantly higher in the combined treatment subgroup, while the SDAI, IL-8 and VEGF values, although higher, did not achieve statistical significance. In both subgroups the SDAI scores decreased significantly at 6 months, as did CRP, ACPA and IL-8 levels, while IL-7, IL-10 and VEGF concentrations also decreased significantly post-therapy in the combined treatment group. The median CRP value post-therapy remained significantly higher in the combined treatment subgroup relative to the MTX monotherapy group (SDAI response in the different treatment groups is shown in Additional file 1: Table S4).

**Table 3:** Subgroup analysis of changes in SDAI values and circulating biomarkers following 6 months DMARD, for the methotrexate (MTX) monotherapy and methotrexate- prednisone combination therapy groups

Variable	n	Baseline		6 months		P value
MTX only		Median	IQR	Median	IQR	
SDAI	43	33.58	24.75	16.03	11.05	0.0001
CRP (μg/ml)	43	9.90	21.30	6.90	13.40	0.0048
ACPA (IU/ml)	36	501.60	844.50	310.90	559.60	0.0033
IL-7 (pg/ml)	42	14.78	64.04	14.17	19.73	NS
IL-8 (pg/ml)	42	7.68	8.84	5.01	5.32	0.0091
IL-10 (pg/ml)	7	6.68	48.16	8.41	23.41	NS
VEGF (pg/ml)	42	114.16	240.82	97.52	184.54	NS
Variable	n	Baseline		6 months		P value
MTX PRED		Median	IQR	Median	IQR	
SDAI	33	38.90	20.64	18.00	18.73	0.0001
CRP (μg/ml)	33	35.90	36.50	11.60	19.10	0.0005
ACPA (IU/ml)	29	598.90	1036.60	367.20	699.50	0.0001
IL-7 (pg/ml)	28	46.67	161.13	21.46	31.06	0.0026
IL-8 (pg/ml)	28	10.35	13.27	5.84	4.03	0.0002
IL-10 (pg/ml)	14	12.99	18.55	8.50	23.95	0.048
VEGF (pg/ml)	28	318.91	482.44	77.08	159.38	0.0044

### Assessment of statistically significant biomarker responses to therapy

These results are shown in Table 4. Subgroup stratification of data by SDAI categories at 6 months, into LDA, MDA and HDA, showed that notable decreases in ACPA levels occurred in all 3 categories. However, statistical significance was evident in only the LDA and MDA subgroups, possibly due to a smaller number of patients in the HDA subgroup. With respect to differences at baseline, the median ACPA value for the LDA subgroup was significantly higher than that of the HDA subgroup (p < 0.034). In the case of the cytokines and chemokines, the most notable decreases at 6 months were observed in the LDA subgroup, with significant decreases in IL-7, IL-8, G-CSF and VEGF concentrations, all of which remained elevated in the HDA subgroup, with the exception of IL-7. No statistical difference could be shown in steroid use between the 3 SDAI categories at 6 months. Patients in the LDA subgroup had a statistically significantly lower SDAI at baseline than the corresponding value for the MDA and HDA subgroups combined (*p* = 0.0070).

**Table 4:** Significant changes in circulating biomarker concentrations, stratified at 6 months

SDAI category ≤ 11						
Variable	n	Baseline		6 months		P value
		Median	IQR	Median	IQR	
SDAI	43	32.42	24.71	6.40	5.34	0.0001
CRP (μg/ml)	43	9.50	20.80	6.90	8.10	0.0054
ACPA (IU/ml)	30	936.55	1162.70	344.60	671.80	0.0005
IL-4 (pg/ml)	9	7.50	7.30	1.50	4.00	0.0280
IL-7 (pg/ml)	36	22.22	82.20	16.19	16.74	0.0050
IL-8 (pg/ml)	36	8.05	11.48	5.56	6.15	0.0169
IL-10 (pg/ml)	9	13.30	15.25	6.85	5.64	0.0382
G-CSF (pg/ml)	36	21.99	99.56	5.78	55.94	0.0095
VEGF (pg/ml)	36	154.96	442.84	98.62	158.33	0.0036
SDAI category > 11 ≤ 26						
Variable	n	Baseline		6 months		P value
		Median	IQR	Median	IQR	
SDAI	62	42.25	23.39	17.15	6.86	0.0001
CRP (μg/ml)	62	26.50	45.60	8.90	17.00	0.0001
ACPA (IU/ml)	42	483.35	1025.50	252.15	564.70	0.0001
IL-4 (pg/ml)	14	4.08	13.73	2.53	8.47	NS
IL-7 (pg/ml)	55	19.18	61.68	18.17	26.61	NS
IL-8 (pg/ml)	55	8.94	8.92	5.85	6.99	0.0029
IL-10 (pg/ml)	14	14.07	39.13	19.18	19.91	NS
G-CSF (pg/ml)	55	9.45	42.11	7.23	51.61	NS
VEGF (pg/ml)	55	168.10	420.05	84.30	169.40	0.0102
SDAI Category >26						
Variable	n	Baseline		6 months		P value
		Median	IQR	Median	IQR	
SDAI	35	43.00	28.50	36.21	11.91	NS
CRP (μg/ml)	35	13.00	44.30	17.00	24.40	NS
ACPA (IU/ml)	28	353.60	917.00	177.00	714.40	NS
IL-4 (pg/ml)	9	1.24	7.18	0.62	1.0	NS
IL-7 (pg/ml)	32	46.67	113.17	13.66	32.18	0.0301
IL-8 (pg/ml)	32	8.55	27.69	6.37	14.49	NS
IL-10 (pg/ml)	9	5.76	8.93	5.81	2.97	NS
G-CSF (pg/ml)	32	11.7	39.12	9.87	54.15	NS
VEGF (pg/ml)	32	170.78	484.05	126.02	187.48	NS

### Associations of genotype with SDAI and circulating biomarker status pre- and post-therapy

These results are shown in Table 5. Analysis of patients stratified by risk allele status revealed a significantly higher (*p* = 0.0452) pre-therapy circulating APCA level in the risk allele-positive group, with no differences between those of the other measured biomarkers, as well as SDAI in the low- and high-risk allele subgroups. Following therapy, SDAI scores and CRP and ACPA concentrations decreased significantly in both the risk allele-negative and -positive subgroups. Falls in the concentrations of circulating cytokines IL-4, IL-8, IL-10, G-CSF and VEGF (*p* = 0.0442 – 0.0001) were noted only in the high-risk allele subgroup, however more patients were on steroids in this subgroup (*p* = 0.0368). (SDAI response in the different risk allele groups is shown in Additional file 1: Table S5).

**Table 5:** Changes in SDAI scores and circulating biomarkers, stratified by shared epitope (risk allele) status

Risk allele negative						
Variable	n	Baseline		6 months		P value
		Median	IQR	Median	IQR	
SDAI	47	39.18	23.50	18.00	19.50	0.0001
CRP (μg/ml)	47	18.00	43.60	7.00	14.60	0.0002
ACPA (IU/ml)	40	331.90	1006.60	185.60	654.15	0.0032
IL-4 (pg/ml)	10	2.55	7.99	5.24	7.18	NS
IL-8 (pg/ml)	44	7.68	14.15	5.23	5.98	NS
IL-10 (pg/ml)	10	7.34	27.79	19.18	19.91	NS
G-CSF (pg/ml)	44	14.42	36.94	12.18	81.23	NS
VEGF (pg/ml)	44	130.52	512.22	92.22	148.07	NS
Risk allele positive						
Variable	n	Baseline		6 months		P value
		Median	IQR	Median	IQR	
SDAI	68	40.04	22.12	16.00	16.52	0.0001
CRP (μg/ml)	68	17.50	38.70	10.10	17.50	0.0005
ACPA (IU/ml)	56	561.10	911.05	277.60	677.80	0.0001
IL-4 (pg/ml)	19	5.00	9.80	1.00	1.50	0.0007
IL-8 (pg/ml)	65	9.49	12.15	5.97	6.20	0.0001
IL-10 (pg/ml)	19	11.24	18.78	5.53	6.52	0.0440
G-CSF (pg/ml)	65	8.66	42.11	1.85	24.74	0.0442
VEGF (pg/ml)	65	190.43	401.81	95.76	160.99	0.0056

On multivariate analysis the changes in the post-therapy concentrations of ACPA, IL-7 and G-CSF (p < 0.05), may have been influenced by the presence of rheumatoid nodules. However, none of the baseline biomarkers evaluated was shown to be predictive of disease activity at 6 months.

## Discussion

Successful therapy with biologic agents in early and established RA has been associated with decreases in circulating ACPA and pro-inflammatory cytokine levels though not always correlating with clinical indices of disease activity [2–13]. However, very few studies have addressed this issue in the therapeutic setting of synthetic DMARDs in early RA, a situation which is likely to be most important in resource-limited settings, including sub-Saharan Africa.

In the present study, ACPA levels decreased significantly post-therapy in the overall cohort of RA patients, as well as in the various sub-groups categorized according to type of therapy (MTX monotherapy or MTX in combination with prednisone), magnitude of post-therapy SDAI category, or risk allele status. The exception was the HDA subgroup in which changes in ACPA and CRP did not achieve statistical significance, possibly due to sustained high disease activity, or a smaller number of patients in the case of ACPA. These findings are in agreement with an earlier study of 66 RA patients which reported decreases in circulating ACPA in a subgroup of early RA patients treated with various synthetic DMARDs, but which did not correlate with either treatment response or type of therapeutic agent [9]. In a more recent study, the absence of a correlation between alterations in ACPA and clinical response following treatment with MTX was also noted in a group of early arthritis patients, although in contrast to the present study, baseline levels of ACPA were found to be predictive of response to MTX [12]. However, the observation in the current study that the median pre-therapy ACPA value was highest in the subgroup of patients that achieved LDA, appears to underscore the lack of a clear relationship between ACPA levels and response to therapy.

ACPA seroreversion following therapy occurred in only a few patients (*n* = 8) as reported by others [31], while a total of 3 patients seroconverted. Although ACPA levels decreased in the majority of patients, an increase was observed in others (*n* = 11, 14.2 %). Although not shown, no differences in SDAI responses were noted between those patients with either increased or decreased levels of ACPA post-DMARD therapy.

Pooled analysis of risk allele-positive (S2/S3P) and -negative (S1/S3D/X) patients revealed a significantly lower baseline ACPA titre in the latter group. Previous studies have described higher circulating concentrations of ACPA in RA patients positive for HLA-DRB*0104 relative to those in patients positive for HLA-DRB*0103, while the S2 and S3P risk alleles have also been reported to be associated with increased levels of ACPA [32–34].

The association between smoking and predisposition for development of seropositive RA is well recognized [35]. However, baseline ACPA levels, as well as the magnitudes of the post-therapy decrements thereof, did not differ significantly between smokers and non-smokers in the present study.

Taken together, the aforementioned findings clearly document significant decreases in circulating ACPA concentrations following 6 months of therapy with synthetic DMARDs, mostly MTX, without or with prednisone, irrespective of baseline SDAI, SE or smoking status. However, the lack of a compelling correlation with changes in clinical indices of disease activity appears to exclude the utility of serial measurement of ACPA as a strategy to monitor the efficacy of therapy with these agents over the initial 6 month period. In contrast, other studies based on therapy with biologic DMARDs such as adalimumab, rituximab and infliximab reported that measurement of ACPA has the potential to guide therapy with these agents [36–39]. Importantly, and not addressed in the present study, ACPA status, as well as alterations in ACPA during the course of RA, may have prognostic value as these have been associated with both disease severity and radiographic progression [40–49]. Moreover, ACPA levels have also been found to correlate with diastolic dysfunction and reduced myocardial mass in patients with RA [50, 51].

With respect to circulating cytokines/chemokines, these generally declined significantly in the entire cohort of patients, as did the IL-1β/IL-1Ra and IL-17/IL-10 ratios. However, as reported by others, alterations in these parameters were not significantly correlated with improvements in clinical indices of disease activity following treatment of RA patients for 6 months with the combination of MTX and prednisone [52]. Increases in the levels of IL-8 following treatment with MTX, which were noted in 30 % of patients in the present study, have been shown to be associated with accelerated radiographic progression [53].

With respect to subgroup analyses, comparison between the MTX monotherapy and MTX + prednisone subgroups revealed significant decreases in IL-7, IL-8, IL-10, and VEGF in the latter subgroup post-therapy, but only IL-8 in the former, possibly supporting the role of low-dose corticosteroids in the management of early RA. Interestingly, pre-therapy IL-7 and CRP levels, which were unknown to the attending clinicians, were significantly higher in those patients earmarked to receive combined therapy, possibly consistent with a perception of more severe disease, perhaps influenced by the increased SJC noted in the patients who received MTX and prednisone.

In the various subgroups categorized according to the magnitude of the SDAI at 6 months, the concentrations of IL 4, IL-7, IL-8, IL-10, G-CSF and VEGF decreased significantly in the LDA subgroup post-therapy , while IL-8 and VEGF, but only IL-7, declined in the MDA and HDA subgroups, respectively. Persistently elevated levels of IL-8, G-CSF and VEGF in the HDA subgroup may implicate neutrophilic inflammation in the immunopathogenesis of more severe disease. However, the lack of correlation between circulating concentrations of the individual cytokines with therapy-associated alterations in disease activity appears to exclude both baseline and serial measurement thereof as a strategy to monitor the success of treatment with synthetic DMARDs. As with ACPA, this contention contrasts with the apparent utility of baseline measurement of cytokines such as IL-6, IL-8, CCL2 and/or TNF as predictors of response to biologic DMARDs, including golimumab, etanercept and infliximab [16, 54–58].

Differences between cytokine profiles in the risk allele-negative and –positive subgroups were also detected post-therapy, possibly reflecting different inflammatory mechanisms and responses to therapy, although significantly more patients were on steroids in the risk allele subgroup. Nonetheless, neither genotype nor any of the circulating biomarkers were found to be predictive of response to therapy.

Potential limitations of the study include firstly, the fairly small numbers of patients in some of the subgroups, especially the HDA subgroup, while the presence of nodulosis and lack of treatment randomization may have influenced the analyses. Secondly, 6 months might be too short a period in which to detect associations of circulating biomarkers of disease activity with clinical responses to therapy. In defence of this strategy however, it has recently been reported that patients who manifest a poor response to traditional DMARDs following 6 months of therapy are unlikely to show further improvement after 12 months [27].

On the other hand, the strengths of the study include: i) the demonstration of the apparent limitations of the measured circulating biomarkers in predicting responses to therapy with synthetic DMARDs in RA patients with relatively early disease; ii) HDA post-therapy is associated with persistently elevated levels of VEGF, IL-8 and G-CSF; iii) combining MTX with oral corticosteroids appears to be associated with a more robust decline in cytokine levels; and iv) ACPA levels measured pre-therapy are significantly higher in patients with the risk allele genotype.

## Conclusions

Our findings show that synthetic DMARD therapy for 6 months is associated with a significant decrease in ACPA and pro-inflammatory cytokines, in tandem with therapeutic response. However, the lack of correlation with measures of disease activity precludes serial measurement of these biomarkers as a strategy to monitor early responses to therapy with synthetic DMARDs. However, the long-term prognostic value of these biomarkers, especially in relation to radiographic progression and functional disability, remains to be established, as does their potential to guide future cost-effective therapies, of particular importance in resource-limited settings.

## Additional file

Additional file 1: Table S1. SDAI (Simplified disease activity index) base line disease activity. **Table S2.** changes in SDAI and circulating biomarker concentrations following 6 months of synthetic DMARD therapy. **Table S3.** SDAI (Simplified disease activity index) response at 6 months. **Table S4.** SDAI (Simplified disease activity index) response at 6 months treatment sub-groups. **Table S5.** SDAI (Simplified disease activity index) response at 6 months risk allele sub-groups.
