# Supplementary material for: Circulating anti-citrullinated peptide antibodies, cytokines and genotype as biomarkers of response to disease-modifying antirheumatic drug therapy in early rheumatoid arthritis
Source: BMC Musculoskelet Disord. 2015 May 29;16:130. doi: 10.1186/s12891-015-0587-1 (PMC4446850; doi:10.1186/s12891-015-0587-1)
Supplement: Supplementary file 1 — SDAI (Simplified disease activity index) base line disease activity. Table S2. changes in SDAI and circulating biomarker concentrations following 6 months of synthetic DMARD therapy. Table S3. SDAI (Simplified disease activity index) response at 6 months. Table S4. SDAI (Simplified disease activity index) response at 6 months treatment sub-groups. Table S5. SDAI (Simplified disease activity index) response at 6 months risk allele sub-groups. [file 12891_2015_587_MOESM1_ESM.pdf]

Additional File

Table S1: SDAI (Simplified disease activity index) base line disease activity

Category	n	Median	IQR
Mild ≤11	3	5.73	5.01
Moderate >11≤26	25	20.00	8.52
High >26	112	45.31	19.47

Table S2: changes in SDAI and circulating biomarker concentrations following 6 months of synthetic DMARD therapy.

Variable	n	median	min	max	iqr	*p* value
SDAI b*	140	41.39	5.28	77	23.67	
SDAI 6**	140	16	0.1	69.77	15.805	0.0001
CRP b	140	17.1	1	303	42.25	
CRP 6	140	8.9	1	83.9	17.35	0.0001
ACPA b	100	516.6	1.9	2526.8	1027.75	
ACPA 6	100	255.65	1.1	1420.3	677.2	0.0001
IL-1 β b	51	6.23	0.12	191.32	13.69	
IL-1 β 6	51	3.8	0.35	199.74	18.17	NS
IL-1Ra b	123	80.06	0	5012.27	288.51	
IL-1Ra 6	123	80.54	0	12084.64	427.81	NS
IL-2 b	32	0	0	216.91	7.41	
IL-2 6	32	0	0	267.6	13.88	NS
IL-4 b	32	4.7	0	904.79	10.28	
IL-4 6	32	1.42	0	62.61	5.31	0.0075
IL-6 b	123	24.35	0	791.93	67.93	
IL-6 6	123	14.23	0	778.82	51.89	NS
IL-7 b	123	20.32	0	6825.1	82.22	
IL-7 6	123	16.69	0	3596	23.46	0.0013
IL-8 b	123	8.79	0	36698.94	12.65	
IL-8 6	123	5.7	0	1618.59	7.09	0.0001
IL-10 b	32	11.435	0	1172.06	29.09	
IL-10 6	32	8.145	1.8	1111	21.71	NS
IL-12 b	123	11.93	0.63	3557.84	64.8	
IL-12 6	123	17.75	0.41	8144.25	74.79	NS

IL-17 b<br>IL-17 6	32<br>32	0<br>0	0<br>0	23.9<br>13.87	0<br>0	 NS
G-CSF b<br>G-CSF 6	123<br>123	13.82<br>7.23	0<br>0	4817.44<br>1328.97	60.02<br>51.61	 0.0083
GM-CSF b<br>GM-CSF 6	123<br>123	1.08<br>0	0<br>0	1458.59<br>2571.87	44.37<br>82.37	 NS
IFNγ b<br>IFNγ 6	123<br>123	42.64<br>27.19	0<br>0	11151.66<br>6394.31	264.72<br>444.72	 NS
TNF b<br>TNF 6	123<br>123	11.67<br>12.15	0.55<br>1.33	2951.68<br>3107.1	69.55<br>93.82	 NS
VEGF b<br>VEGF 6	123<br>123	168.56<br>92.62	0<br>0	4503.15<br>5007.35	441.33<br>167.21	 0.001
CCL-2 b<br>CCL-2 6	32<br>32	10.65<br>50.65	0<br>0	521.48<br>566.52	100.955<br>81.59	 NS
CCL-4 b<br>CCL-4 6	32<br>32	100.905<br>96.275	26.67<br>27.26	344.72<br>326.37	91.10001<br>62.46	 NS
IL-1/IL1-ra b<br>IL-1/IL-1ra 6	37<br>37	0.050012<br>0.0282269	0.0057609<br>0.0029457	0.9042904<br>0.4552684	0.0487324<br>0.0622231	 0.013
IL-17/IL-10 b<br>IL-17/ IL-10 6	30<br>30	10.41581<br>3.723691	0.3762998<br>0.6393507	118.3059<br>22.96679	15.60833<br>5.373202	 0.0001
IFNg/ IL-4 b<br>IFNg/ IL-4 6	24<br>24	25.17977<br>21.07841	0<br>1.105882	179.2<br>83.55289	28.12688<br>33.04997	 NS
IL-8/ IL-10 b<br>IL-8/ IL-10 6	30<br>30	0.8005226<br>0.796825	0.0097521<br>0.0068947	45.19495<br>27.28358	2.059342<br>1.268058	 NS
IL-6/ IL-10 b<br>IL-6/ IL-10 6	30<br>30	1.046355<br>1.834637	0<br>0	33.66154<br>37.93333	2.474186<br>6.346017	 NS

*b – baseline *6 – 6 months *NS – not significant
SDAI, Simplified disease activity index; CRP, C-reactive protein; ACPA, Anti-cyclic citrullinated peptide antibodies; IL-, Interleukin; G-CSF, Granulocyte colony-stimulating factor; GM-CSF, Granulocyte and macrophage colony-stimulating factor; IFN, Interferon; TNF, Tumour necrosis factor; VEGF, Vascular endothelial growth factor; CCL-, Chemokine (C-C motif) ligand-.

Table S3: SDAI (Simplified disease activity index) response at 6 months

Response		n
< 50%	(no response)	58
≥50%<70%	(minor response)	40
≥70%<85%	(moderate response)	22
≥ 85%	(major response)	20

Table S4: SDAI (Simplified disease activity index) response at 6 months treatment sub-groups

Response		n (Methotrexate plus prednisone)	n (Methotrexate only)
< 50%	(no response)	14	16
≥50%<70%	(minor response)	12	14
≥70%<85%	(moderate response)	4	8
≥ 85%	(major response)	3	5

Table S5: SDAI (Simplified disease activity index) response at 6 months risk allele sub-groups

Response		n (risk allele positive)	n (risk allele negative)
< 50%	(no response)	27	21
≥50%<70%	(minor response)	21	13
≥70%<85%	(moderate response)	11	6
≥ 85%	(major response)	9	7
